# *Propionibacterium acnes* Infection after Craniotomy in a Young Adult with Subdural Hematoma

**DOI:** 10.1155/2021/6695286

**Published:** 2021-02-03

**Authors:** Hind El Soufi, Yahya El Soufi, Farshad Bagheri

**Affiliations:** ^1^Jamaica Hospital Medical Center, Internal Medicine Department, 8900 Van Wyck Expressway, Richmond Hill, NY 11418, USA; ^2^Lebanese University, Faculty of Medical Sciences, Beirut, Lebanon; ^3^Jamaica Hospital Medical Center, Director of Infectious Diseases Department, Richmond Hill, NY 11418, USA

## Abstract

*Propionibacteriumacnes* is a Gram-positive bacillus that can be part of the human skin flora. This bacterium infecting the subdural space postoperatively is quite a rare entity. When present, it likely reflects a true infection rather than contamination requiring urgent intervention. We are reporting a rare case of craniotomy for subdural hematoma evacuation complicated by subdural *Propionibacterium acnes* infection. The infection was successfully treated with surgical debridement and antibiotics.

## 1. Introduction

*Propionibacterium acnes  *(*P. acnes*), currently known as *Cutibacterium acnes* (*C. acnes*, is a slow-growing nonsporulating anaerobic Gram-positive bacillus that is usually considered part of the human skin flora especially the scalp [1]. It can also be present in sebaceous glands and hair follicles which makes it difficult to determine if the positive culture is reflecting a true infection or a contamination [2]. *P. acnes* is usually more likely to be pathogenic when growing in more than one specimen or when cultured from a deep intraoperative specimen [3]. Furthermore, postcraniotomy infection caused by *P. acnes* is relatively uncommon, but when it occurs, it can be associated with significant complications and can become life-threatening [4]. Only few cases have been reported in the literature [5]. In this case report, we present a rare case of craniotomy with subdural hematoma (SDH) evacuation complicated by subdural *P. acnes* infection in a young male patient.

## 2. Case Presentation

**Figure 1:**
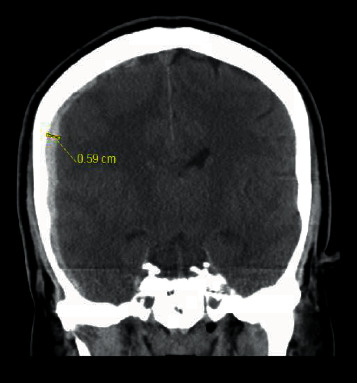
Noncontrast coronal CT head showing right crescent-shaped subdural blood collection measuring up to 6 mm in thickness.

**Figure 2:**
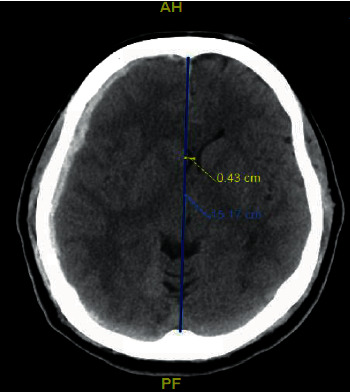
Noncontrast axial CT head showing the right subdural hematoma with right to left midline shift of 4 mm.

**Figure 3:**
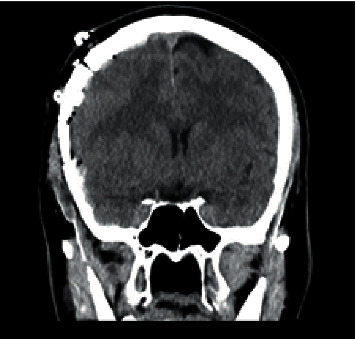
Noncontrast coronal CT head status after right frontoparietal craniotomy with subdural drain placement showing the evacuated right subdural hematoma with presence of residual-mixed density fluid and air.

A 42-year-old man with a history of well-controlled hypertension presented to the hospital complaining of progressively worsening headaches associated with dizziness and blurry vision for three days. The patient denied any history of fever, chills, vomiting, head trauma, or loss of consciousness. He also denied any history of alcohol, vitamins, or anabolic drug use. Upon presentation, the patient was hemodynamically stable with normal physical and neurological examinations. All blood tests were within normal limits. CT head without contrast revealed acute right crescent-shaped subdural blood collection measuring up to 6 mm in thickness with mild falx herniation and right to left midline shift of 4 mm (Figures 1 and 2). CT cerebral angiogram showed no aneurysm and no active bleeding within the hematoma with prominent cortical veins on the right peri-sylvian region. CT angiogram of the neck revealed no abnormalities, and CT cervical spine reported no evidence of acute spine fracture. Diagnostic angiogram revealed no evidence of vascular malformation. The patient underwent a successful right craniotomy for evacuation of his atraumatic acute right-sided SDH with coagulation of the bleeding veins and placement of a subdural drain and a bone flap to the skull. A postoperative CT head showed successful evacuation of the SDH (Figure 3). The patient remained hemodynamically and neurologically stable. The drain was removed, and the patient was then discharged home with outpatient follow up plan.

However, one week later, the patient returned to the hospital complaining of persistent dizziness and headaches not improving with pain medications. A repeat CT head showed mixed density right hemispheric SDH that has increased in size with increased mass effect and midline shift suggestive of chronic reaccumulating SDH. Neurosurgery team recommended a repeat CT head after 24 hours or earlier if any change in his neurological exam. CT head was subsequently done and showed stable hematoma with mild decreased midline shift for which the patient was started on steroids and ultimately discharged home with follow-up in outpatient clinics.

**Figure 4:**
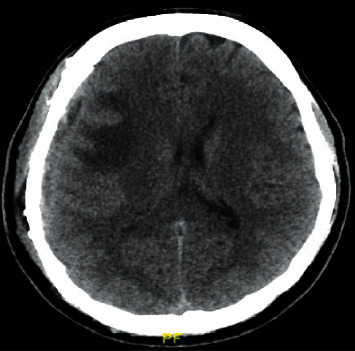
Noncontrast axial CT head showing a new area of diminished attenuation in the right frontal lobe suggestive of vasogenic edema.

**Figure 5:**
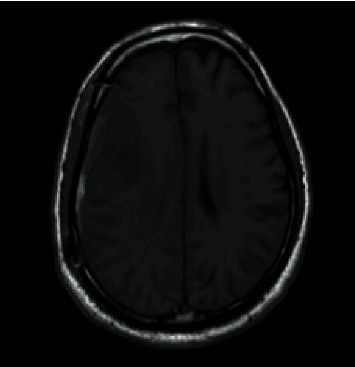
Axial T1-weighted MRI brain showing a subdural collection with mixed density along the right convexity.

**Figure 6:**
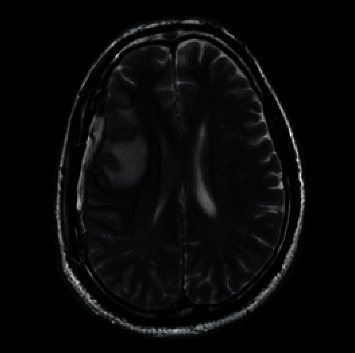
Axial T2-weighted MRI brain showing a subdural collection with mixed density along the right convexity.

**Figure 7:**
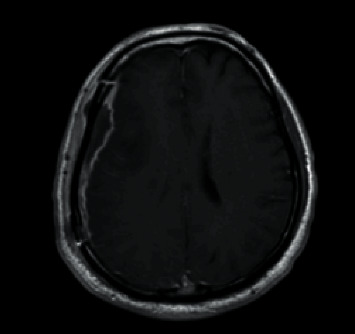
Axial postcontrast T1-weighted MRI brain showing a subdural collection along the right convexity measuring up to 0.9 cm in thickness.

Two weeks after discharge, the patient started reexperiencing severe headaches with subjective fever of one-day duration for which the patient returned to the hospital. In ER, the patient was febrile with normal vital signs. Blood test was significant for leukocytosis with neutrophilic shift and elevated CRP and ESR. CT head without contrast revealed decrease in size of previous right SDH with a new area of diminished attenuation in the right frontal lobe suggestive of vasogenic edema (Figure 4). MRI brain was performed confirming the CT findings with presence of right subdural empyema (Figures 5–7). The patient underwent a right craniectomy for removal of previously placed bone plate and for exploration washout of the subdural collection. During the procedure, a yellowish purulent material was seen in the subdural space from which multiple specimen swabs were taken and sent for culture. The subdural space was well debrided. The patient was started on broad-spectrum antibiotics with intravenous vancomycin, cefepime, and metronidazole, awaiting cultures result. The previous bone flap was not placed back due to underlying infection. Swab culture remained negative for 96 hours before growing *P. acnes* bacteria. Antibiotics were then de-escalated to aqueous crystalline penicillin for a total treatment duration of 6 weeks.

Two months later, after completion of the full course of antibiotics and during a follow-up visit to the infectious disease clinic, the patient reported improvement of symptoms. Repeat blood test was negative for any sign of persistent infection. Appointment was then set with neurosurgery office for cranioplasty scheduling.

## 3. Discussion

Subdural hematoma is defined as the presence of blood between the dura and the arachnoid membranes. It is most commonly a result of a traumatic brain injury that occurs often in the elderly [6]. At younger ages, spontaneous SDH is very unusual but when it occurs, the symptoms are known to be headaches in 59.5% and seizures in 21.4% [7]. In a review of literature done by Hesselbrock et al. that included 21 young patients with no known history of previous head trauma, the main risks factors for spontaneous SDH were elevated blood pressure, chronic alcohol use, infections, arteriovascular malformations, malignancies, and coagulopathies [8]. In our case, it is proposed that the SDH was related to the patient's history of hypertension since the patient did not have any of the other abovementioned risk factors. On the other hand, infected subdural hematoma is considered a rare condition. In a review of literature done by Yamamoto et al. in 2015, only 27 cases of infected SDH were reported [5]. Most infections were caused by methicillin-resistant *Staphylococcus aureus*, *Escherichia coli*, *Salmonella*, and *Klebsiella* species [9, 10]. *P. acnes* bacterium was rarely reported in the literature to cause infection in the subdural space. Cultures positive for *P. acnes* may sometimes represent a false-positive result due to contamination which makes the diagnosis of a true infection often challenging [11]. Moreover, these organisms may be difficult to identify initially because of their slow growth. When cultured, they often need more than three days to grow. In addition, *P. acnes* may be overlooked when cultures are not rechecked or if cultures are discarded after three or five days of incubation. In a study done by Frangiamore et al. in 2015, the time to positive culture appeared to be shorter among patients with true infection with a median time of five days versus nine days among patients with probable contamination [12]. In our case, fever, leukocytosis, neutrophilia, positive inflammatory markers, and subdural empyema found after craniotomy were all clinical indicators of a true infection and *P. acnes* grew after four days of culture. An effective antibiotic regimen includes at least two weeks of parenteral therapy, with penicillin being the drug of choice [13, 14]. Therapy is usually followed by an oral *β*-lactam agent such as a first-generation oral cephalosporin for a minimum duration of two weeks [15].

In summary, while *P. acnes* infection is usually not pathogenic, it can sometimes be very challenging for physicians to differentiate between a true infection and a simple contamination that does not require treatment. Hence, keeping a high clinical suspicion for infection and shorter time to positive culture can always be clues in stratifying these patients. On the other hand, *P. acnes* infecting the subdural space postoperatively is quite rare, but when this bacterium is isolated from the subdural space, it most likely reflects a true infection that requires to be immediately treated by proper debridement and antibiotic coverage. Finally, taking multiple specimens from the site of infection, rechecking the cultures, and avoiding early discard of the specimens are very important.
